# Mining patents with large language models elucidates the chemical function landscape†

**DOI:** 10.1039/d4dd00011k

**Published:** 2024-05-07

**Authors:** Clayton W. Kosonocky, Claus O. Wilke, Edward M. Marcotte, Andrew D. Ellington

**Affiliations:** a Department of Molecular Biosciences, University of Texas at Austin Austin TX 78705 USA; b Department of Integrative Biology, University of Texas at Austin Austin TX 78705 USA; c Center for Systems and Synthetic Biology, University of Texas at Austin Austin TX 78705 USA

## Abstract

The fundamental goal of small molecule discovery is to generate chemicals with target functionality. While this often proceeds through structure-based methods, we set out to investigate the practicality of methods that leverage the extensive corpus of chemical literature. We hypothesize that a sufficiently large text-derived chemical function dataset would mirror the actual landscape of chemical functionality. Such a landscape would implicitly capture complex physical and biological interactions given that chemical function arises from both a molecule's structure and its interacting partners. To evaluate this hypothesis, we built a Chemical Function (CheF) dataset of patent-derived functional labels. This dataset, comprising 631 K molecule–function pairs, was created using an LLM- and embedding-based method to obtain 1.5 K unique functional labels for approximately 100 K randomly selected molecules from their corresponding 188 K unique patents. We carry out a series of analyses demonstrating that the CheF dataset contains a semantically coherent textual representation of the functional landscape congruent with chemical structural relationships, thus approximating the actual chemical function landscape. We then demonstrate through several examples that this text-based functional landscape can be leveraged to identify drugs with target functionality using a model able to predict functional profiles from structure alone. We believe that functional label-guided molecular discovery may serve as an alternative approach to traditional structure-based methods in the pursuit of designing novel functional molecules.

## Abstract 2

This study creates a chemical function dataset by using an LLM to extract information from patents. Analyses show the dataset is semantically structured, coheres with structural relationships, and can be used to train structure-to-function models.

## Graphical abstract

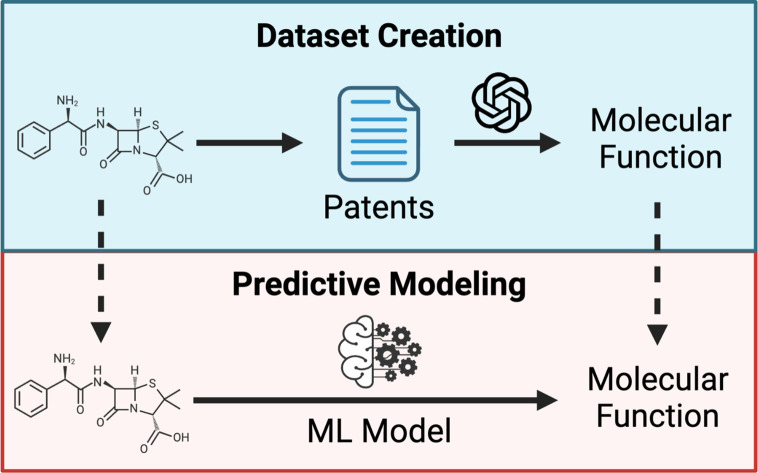

## 1. Introduction

The overarching goal of drug discovery is to generate chemicals with specific functionality through the design of chemical structure.^1^ Functionality, often in the context of drug discovery, refers to the specific effects a chemical exhibits on biological systems (*i.e.*, vasodilator, analgesic, protease inhibitor), but it is applicable to materials as well (*i.e.*, electroluminescent, polymer). Computational methods often approach molecular discovery through structural and empirical methods such as protein–ligand docking, receptor binding affinity prediction, and pharmacophore design.^2–5^ These methods are powerful for designing molecules that bind to specific protein targets, but at present they are unable to explicitly design for specific organism-wide effects. This is largely because biological complexity increases with scale, and many whole-body effects are only weakly associated with specific protein inhibition or biomolecular treatment.^6^

Humans have long been documenting chemicals and their effects, and it is reasonable to assume functional relationships are embedded in language itself. Text-based functional analysis has been paramount for our understanding of the genome through Gene Ontology terms.^7^ Despite its potential, text-based functional analysis for chemicals has been largely underexplored. This is in part due to the lack of high-quality chemical function datasets but is more fundamentally due to the high multi-functionality of molecules, which is less problematic for genes and proteins. High-quality chemical function datasets have been challenging to generate due to the sparsity and irregularity of functional information in chemical descriptions, patents, and literature. Recent efforts at creating such datasets tend to involve consolidation of existing curated descriptive datasets.^8–12^ Similarly, keyword-based function extraction partially solves the function extraction problem by confining its scope to singular predetermined functionality, but it fails at broadly extracting all relevant functions for a given molecule.^13^ Given their profound success in text summarization, Large Language Models (LLMs) may be ideal candidates to broadly extract functional information of molecules from patents and literature, a task that remains underexplored.^14–16^ This is especially promising for making use of the chemical patent literature, an abundant and highly specific source of implicit chemical knowledge that has been largely inaccessible due to excessive legal terminology.^17,18^ LLMs have been used in this way to help evaluate functional relevance of results from a machine learning-based chemical similarity search.^19^ This may allow for the creation of a large-scale dataset that effectively captures the text-based chemical function landscape.

We hypothesize that a sufficiently large chemical function dataset would contain a text-based chemical function landscape congruent with chemical structure space, effectively approximating the actual chemical function landscape. Such a landscape would implicitly capture complex physical and biological interactions given that chemical function arises from both a molecule's structure and its interacting partners.^20^ This hypothesis is further based on the observation that function is reported frequently enough in patents and scientific articles for most functional relationships to be contained in the corpus of chemical literature.^21^ To evaluate this hypothesis, we set out to create a Chemical Function (CheF) dataset of patent-derived functional labels. This dataset, comprising 631 K molecule–function pairs, was created using an LLM- and embedding-based method to obtain functional labels for approximately 100 K molecules from their corresponding 188 K unique patents. The CheF dataset was found to be of high quality, demonstrating the effectiveness of LLMs for extracting functional information from chemical patents despite not being explicitly trained to do so. Using this dataset, we carry out a series of experiments alluding to the notion that the CheF dataset contains a text-based functional landscape that simulates the actual chemical function landscape due to its congruence with chemical structure space. We then demonstrate through several examples that this text-based functional landscape can be harnessed to identify drugs with target functionality using a model able to predict functional profiles from structure alone. We believe that functional label-guided molecular discovery may serve as an alternative approach to traditional structure-based methods in the pursuit of designing novel functional molecules.

## 2. Methods

### 2.1 Dataset creation

The SureChEMBL database, a database of text-mined associations between molecules and the patents they are mentioned in, was shuffled and converted to chiral RDKit-canonicalized SMILES strings to remove malformed strings.^21–23^ SMILES strings were converted to InChIKeys and used to obtain PubChem CIDs, using the larger CID when conflicting pairs exist.^24^ To minimize costs and prevent label dilution, only molecules with fewer than 10 patents were included. This reduced the dataset from 32M to 28.2M molecules, a 12% decrease. A random 100 K molecules were selected as the dataset. For each associated patent, the title, abstract, and description were scraped from Google Patents and cleaned.

The patent title, abstract, and first 3500 characters of the description were summarized into concise functional labels using ChatGPT (gpt-3.5-turbo) with no further fine-tuning from July 15th, 2023, chosen for low cost and high speed. Cost per molecule was $0.005 using gpt-3.5-turbo. The first 3500 characters of the description were included because the start of the patent description typically contains relevant background, mechanistic information, and/or a summary of the claim. Responses from ChatGPT were converted into sets of labels and linked to their associated molecules. Summarizations were cleaned, split into individual words, converted to lowercase, and converted to singular if plural. Single-character labels were removed. The cleaned dataset resulted in 29 854 unique labels for 99 182 molecules. Fetching patent information and summarizing with ChatGPT, this method's bottleneck, took 6 s per molecule with 16 CPUs in parallel. This could be sped up to 3.9 s by summarizing per-patent rather than per-molecule to avoid redundant summarizations, and even further to 2.6 s by using only US and WO patents.

To consolidate labels by semantic meaning, the vocabulary was embedded with OpenAI's text-embedding-ada-002 and clustered to group labels by embedding similarity. DBSCAN clustering was performed on the embeddings with a sweeping epsilon.^25^ The authors chose the epsilon for optimal clustering, set to be at the minimum number of clusters without quality degradation (*e.g.*, avoiding the merging of antiviral, antibacterial, and antifungal). The optimal epsilon was 0.34 for the dataset herein, consolidating down from 29 854 to 20 030 labels. Representative labels for each cluster were created using gpt-3.5-turbo. The labels from a very large cluster of only IUPAC structural terms were removed to reduce non-generalizable labels. Despite this, some structural terms remained which correspond either to receptor names (*i.e.*, ATP, 5-HT), or to chemical moieties (*i.e.*, aryl, azetidine). Labels appearing in <50 molecules were dropped to ensure sufficient predictive power. Single character labels were then dropped. This resulted in a 99 182-molecule dataset with 1522 unique functional labels, deemed the Chemical Function (CheF) dataset.

### 2.2 ChatGPT patent summarization validation

Manual validation was performed on 200 molecules randomly chosen from the CheF dataset. These 200 molecules had 596 valid associated patents, and 1738 ChatGPT summarized labels. These labels were manually validated to determine the ratio of correct syntax, relevance to patent, and relevance to the molecule of interest.

### 2.3 Validation of ChatGPT-aided label consolidation

The first 500 of the 3178 clusters of greater than one label (sorted in descending cluster size order) were evaluated for whether or not the clusters contained semantically common elements. The ChatGPT consolidated cluster labels were then analyzed for accuracy and representativeness. Common failure modes for clustering primarily included the grouping of grammatically similar, but not semantically similar labels (*e.g.*, ahas-inhibiting, ikk-inhibiting). Failure modes for ChatGPT commonly included averaging the terms to the wrong shared common element (*e.g.*, anti-fungal and anti-mycotic being consolidated to the label “anti”).

### 2.4 Text-based functional landscape graph

Per-molecule label co-occurrence was counted across CheF. Counts were used as edge weights between label nodes to create a graph, visualized in Gephi using force atlas, nooverlap, and label adjust methods (default parameters).^26^ Modularity-based community detection with 0.5 resolution resulted in 19 communities.

### 2.5 Coincidence of labels and their neighbors in structure space

The 99 182 molecules were converted to daylight molecular fingerprints with the RDKfingerprint() method in RDKit.^23^ These fingerprints were t-SNE projected using sckit-learn, setting the perplexity parameter to 500. Molecules were colored if they contained a given label, see https://chefdb.app. The max fingerprint Tanimoto similarity from each molecule containing a given label to each molecule containing any of the 10 most commonly co-occurring labels was computed. The null co-occurrence was calculated by computing the max similarity from each molecule containing a given label to a random equal-sized set. Significance for each label was computed with an independent 2-sided *t*-test. The computed *P* values were then subjected to a false-discovery-rate (FDR) correction and the labels with *P* < 0.05 after FDR correction were considered significantly clustered (Benjamini & Hochberg, 1995). Limiting max co-occurring label abundance to 1 K molecules was necessary to avoid polluting the analysis, as hyper-abundant labels would force the Tanimoto similarity to 1.0.

### 2.6 Model training

Several multi-label classification models were trained to predict the CheF from molecular representations. These models included logistic regression (*C* = 0.001, max_iter = 1000), random forest classifier (*n*_estimators = 100, max_depth = 10), and a multilayer perceptron (BCEWithLogitsLoss, layer sizes (512, 256), 5 epochs, 0.2 dropout, batch size 32, learning rate 0.001; 5-fold CV to determine params). A random 10% test set was held out from all model training. Macro average and individual label ROC-AUC and PR-AUC were calculated.

### 2.7 Patent summarization prompt

For gpt-3.5-turbo, the system prompt used was “You are an organic chemist summarizing chemical patents”, and the user prompt was “Return a short set of three 1–3 word descriptors that best describe the chemical or pharmacological function(s) of the molecule described by the given patent title, abstract, and partial description (giving more weight to title & abstract). Be specific and concise, but not necessarily comprehensive (choose a small number of great descriptor). Follow the syntax ‘{descriptor_1}/{descriptor_2}/{*etc*}’, writing ‘NA’ if nothing is provided. DO NOT BREAK THIS SYNTAX. The following is the patent:”, followed by the patent title, abstract, and partial description.

### 2.8 Word embedding cluster summarization prompt

Each cluster's labels were fed into gpt-3.5-turbo with the system prompt “You are a PhD pharmaceutical chemist” and the user prompt: “Given a set of molecular descriptors, return a single descriptor representing the centroid of the terms. Do not speculate. Only use the information provided. Be concise, not explaining answers. Example 1 set of descriptors: 11(beta)-hsd1, 11-hsd-2, 17β-hsd3 example 1 average descriptor: hsd example 2 set of descriptors: anti-retroviral, anti-retrovirus, anti-viral, anti-virus, antiretroviral, antiretrovirus, antiviral, antivirus example 2 average descriptor: antiviral set of descriptors: __INSERT_DESCRIPTORS_HERE__ average descriptor:”.

### 2.9 Graph label cluster summarization prompt

Each cluster's labels were fed into GPT-4 with the system prompt “You are a PhD pharmaceutical chemist” and the user prompt: “Pretend you are a pharmaceutical chemist. I will provide you with several terms, and your job is to summarize the terms into appropriate categories. Be succinct, focusing on the broadest categories while still being representative. Don't show your work. Example terms: antiviral HCV kinase cancer polymerase protease example summarization: antiviral & cancer terms: __INSERT_DESCRIPTORS_HERE__ summarization:”.

## 3. Results

Patents are an abundant source of highly specific chemical knowledge. It is plausible that a large dataset of patent-derived molecular function would capture most known functional relationships and could approximate the chemical function landscape. High-fidelity approximation of the chemical function landscape would implicitly capture complex physical and biological interactions given that chemical function arises from both a molecule's structure and its interacting partners. This would allow for the prediction of functional labels for chemicals which is, to our knowledge, a novel task.

### 3.1 Chemical function dataset creation

We set out to create a large-scale database of chemicals and their patent-derived molecular functionality. To do so, a random 100 K molecules and their associated patents were chosen from the SureChEMBL database to create a Chemical Function (CheF) dataset (Fig. S1†).^21^ As our goal was to capture molecular functionality in the broadest sense, we chose to include patents irrespective of their International Patent Classification categories (Table S1†). To ensure that patents were highly relevant to their respective molecule, only molecules with fewer than 10 patents were included in the random selection, reducing the number of available molecules by 12%. This was done to exclude over-patented molecules like penicillin with over 40 000 patents, most of which are irrelevant to its functionality (Table S2†). We acknowledge that this filter removes the most well-studied molecules from the dataset. However, we hypothesize that the impact of this holdout is minimal as models trained on the dataset will be able to infer functionality of well-studied molecules from their less-patented derivatives.

**Fig. 1:**
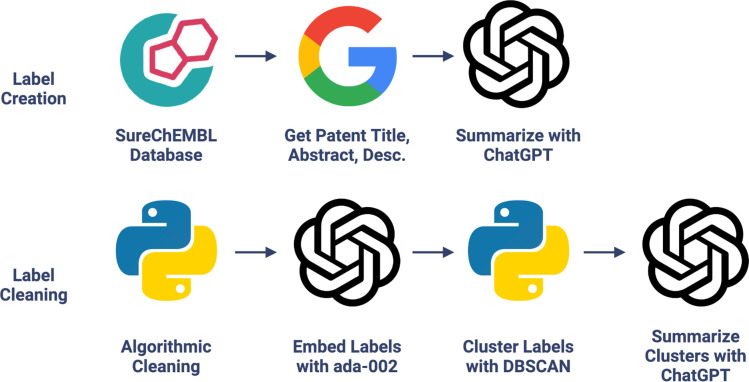
Chemical function dataset creation. LLM extracts molecular functional information present in patents into concise labels; see Fig. S2† for an example. Chemical functional labels were then cleaned with algorithmic-, embedding-, and LLM-based methods.

For each molecule–associated patent in the CheF dataset, the patent title, abstract, and description were scraped from Google Patents and cleaned. ChatGPT (gpt-3.5-turbo) was used to generate 1–3 functional labels describing the patented molecule given its unstructured patent data (Fig. 1 and Table S3†). The LLM-assisted function extraction method's success was validated manually across 1738 labels generated from a random 200 CheF molecules. Of these labels, 99.6% had correct syntax and 99.8% were relevant to their respective patent. In the SureChEMBL database, molecules can be linked to patents in which they serve as intermediates to the final patented molecule. Because of this, 77.9% of the labels correctly describe the labeled molecule's function. However, if considering associations through synthesis, then 98.2% of the molecules are correctly described by their functional labels. This shows that the deviation from near-perfect accuracy is due to the molecule–patent associations rather than the ChatGPT-assisted functional extraction.

The LLM-assisted method resulted in 104 607 functional labels for the 100 K molecules (see Fig. S3† for the top terms). These were too many labels to yield any predictive power, so measures were taken to consolidate these labels into a concise vocabulary. The labels were cleaned, reducing the number of labels to 39 854, and further consolidated by embedding each label with a language model (OpenAI's text-embedding-ada-002) to group grammatically dissimilar yet semantically similar labels together. The embeddings were clustered with DBSCAN using a cutoff that minimized the number of clusters without cluster quality deterioration (*e.g.*, avoiding the grouping of antiviral, antibacterial, and antifungal) (Fig. S4†). Each cluster was summarized with GPT-4 to obtain a single representative cluster label.

The embedding-based clustering and summarization process was validated across the 500 largest clusters. Of these, 99.2% contained semantically common elements and 97.6% of the cluster summarizations were accurate and representative of their constituent labels. These labels were mapped back to the CheF dataset, resulting in 19 616 labels. To ensure adequate predictive power, labels appearing in less than 50 molecules and labels with only a single character were dropped. The final CheF dataset consisted of 99 182 molecules and their 1522 descriptive functional labels. A comparison to similar datasets is made in Table 1, outlining the unique scalability of the CheF dataset.

**Table 1:** Comparison of chemical-text datasets^a^

Dataset	Curr. size	Scaleup size	Text-type	S/F separate	Data source
ChEBI	103 K	103 K+	Long text	No	DB agg./manual
ChemFOnt	342 K	1M+	Labels	Yes	DB agg./manual
CheF (ours)	100 K	32M+	Labels	Yes	LLM-sum. patents

a Comparison of CheF to existing chemical-text datasets ChEBI and ChemFOnt^8,9^ by current size (# molecules), maximum automated scaleup size (# molecules), text-type, whether or not structure and function are separate in the text, and the data source used for dataset construction. Both ChEBI and ChemFOnt were built from existing datasets with additional manual curation and annotation, limiting potential automated scaleup size. In contrast, the method used to build CheF scales readily, allowing for a potential dataset size of 32M + molecules.

### 3.2 Functional labels map to natural clusters in chemical structure space

Molecular function nominally arises directly from structure, and thus any successful dataset of functional labels should cluster in structural space. This hypothesis was based in part on the observation that chemical function is often retained despite minor structural modifications.^27,28^ Due to molecules and their derivatives frequently being patented together, structurally similar molecules should be annotated with similar patent-derived functions. This rationale generally holds, but exceptions include stereoisomers with different functions (*e.g.* as for thalidomide) and distinct structures sharing the same function (*e.g.* as for beta-lactam antibiotics and tetracyclines).

**Fig. 2:**
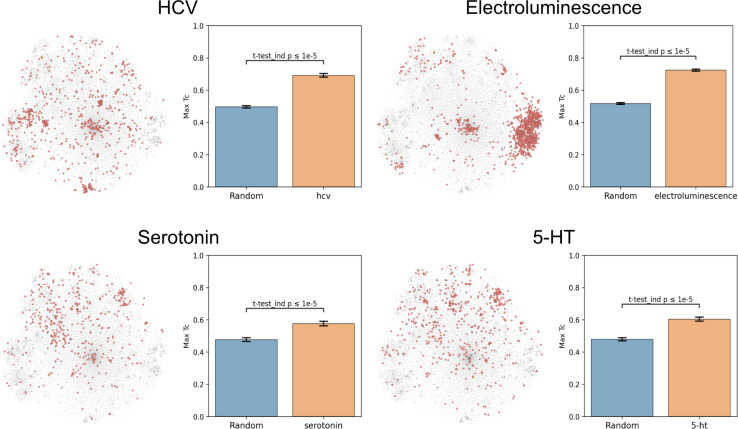
Text-based functional labels cluster in structural space. For each of the labels “hcv”, “electroluminescence”, “serotonin”, and “5-HT”, molecules in the CheF dataset were mapped by their molecular fingerprints and colored based on whether the selected label was present in their set of functional descriptors. The max fingerprint Tanimoto similarity was computed between the fingerprint vectors of each molecule containing a given label and was compared against the max fingerprint Tanimoto similarity from a random equal-sized set of molecules to determine significance to a random control. Many of the labels strongly cluster in structural space, demonstrating that CheF accurately captures structure–function relationships. See Fig. S5† for examples with more labels.

To evaluate this hypothesis, we embedded the CheF dataset in structure space by converting the molecules to daylight molecular fingerprints (binary vectors representing a molecule's substructures), visualized with t-distributed Stochastic Neighbor Embedding (t-SNE) (Fig. 2 and S5†).^23^ Then, to determine if the CheF functional labels clustered in this structural space, the maximum fingerprint Tanimoto similarity was computed between the fingerprint vectors of each molecule containing a given label; this approach provides a measure of structural similarity between molecules that have the same functional label.^29^ This value was compared to the maximum similarity computed from a random equal-sized set of molecules to determine significance. Remarkably, 1261 of the 1522 labels were found to cluster significantly in structural space (independent *t*-tests per label, false-discovery rate of 5%). To give an idea of the meaning of this correlation, inherent clustering was visualized for the labels ‘hcv’ (hepatitis C virus), ‘electroluminescence’, ‘serotonin’, and ‘5-HT’ (5-hydroxytryptamine, the chemical name for serotonin) (Fig. 2). For the label ‘electroluminescence’, there was one large cluster containing almost only highly conjugated molecules. For ‘hcv’, there were multiple distinct communities representing antivirals targeting different mechanisms of HCV replication (Fig. S6†). Clusters were observed for NS5A inhibitors, NS3 macrocyclic and peptidomimetic protease inhibitors, and nucleoside NS5B polymerase inhibitors. The observed clustering of functional labels in structure space provided evidence that the CheF dataset labels had accurately captured structure–function relationships, validating our initial hypothesis.

### 3.3 Label co-occurrences reveal the text-based chemical function landscape

**Fig. 3:**
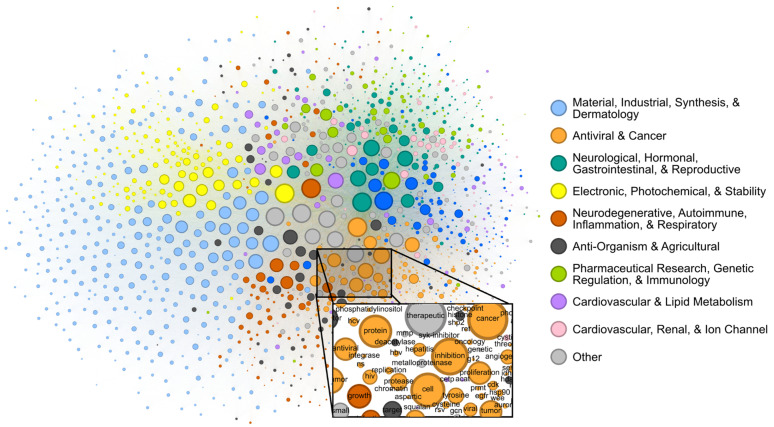
Label co-occurrences reveal the text-based chemical function landscape. Node sizes correspond to number of connections, and edge sizes correspond to co-occurrence frequency in the CheF dataset. Modularity-based community detection was used to obtain 19 distinct communities. The communities broadly coincided with the semantic meaning of the contained labels, the largest 10 of which were summarized to representative categorical labels (Tables S4–S6†).

Patents contain joint contextual information on the application, structure, and mechanism of a given compound. We attempted to determine the extent to which the CheF dataset implicitly captured this joint semantic context by assessing the graph of co-occurring functional labels (Fig. 3). Each node in the graph represents a CheF functional label, and their relative positioning indicates the frequency of co-occurrence between labels, with labels that co-occur more frequently placed closer together. To prevent the visual overrepresentation of extremely common labels (*i.e.*, inhibitor, cancer, kinase), each node's size was scaled based on its connectivity instead of the frequency of co-occurrence.

Modularity-based community detection isolates tightly interconnected groups within a graph, distinguishing them from the rest of the graph. This method was applied to the label co-occurrence graph, with the resulting clusters summarized with GPT-4 into representative labels for unbiased semantic categorization (Tables S4–S6†). The authors curated the summarized labels for validity and found them representative of the constituent labels; these were then further consolidated for succinct representation of the semantic categorization (Table S4†). This revealed a semantic structure in the co-occurrence graph, where distinct communities such as ‘Electronic, Photochemical, & Stability’ and ‘Antiviral & Cancer’ could be observed (Fig. 3). Within communities, the fine-grained semantic structure also appeared to be coherent. For example, in the local neighborhood around ‘hcv’, the labels ‘antiviral’, ‘ns’ (nonstructural), ‘hbv’ (hepatitis B virus), ‘hepatitis’, ‘replication’, and ‘protease’ were found, all of which are known to be semantically relevant to hepatitis C virus (Fig. 3). The graph of patent-derived molecular functions is a visual representation of the text-based chemical function landscape and represents a potentially valuable resource for linguistic evaluation of chemical function and ultimately drug discovery.

### 3.4 Coherence of the text-based chemical function landscape in chemical structure space

**Fig. 4:**
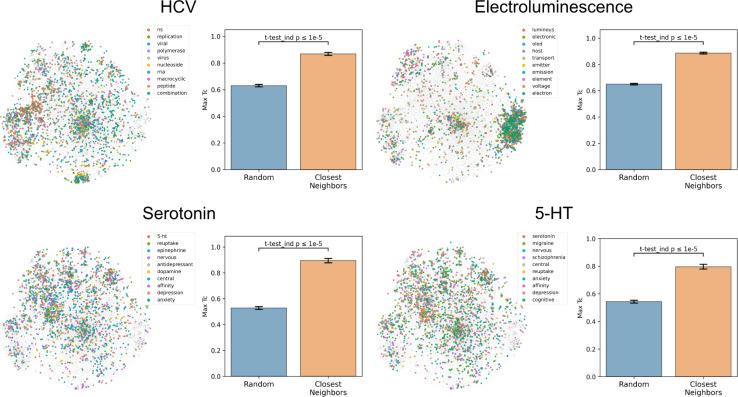
Coherence of the text-based chemical function landscape in structure space. To assess the alignment of text-based functional relationships with structural relationships, for each of the labels “hcv”, “electroluminescence”, “serotonin”, and “5-HT”, the max fingerprint Tanimoto similarity from each molecule containing a given label to each molecule containing any of its 10 most frequently co-occurring labels (<1000 total abundance) was compared against the max fingerprint Tanimoto similarity to a random subset of molecules of the same size. See Fig. S5† for examples with more labels.

To assess how well text-based functional relationships align with structural relationships, the overlap between the molecules of a given label and those of its 10 most commonly co-occurring labels was calculated (Fig. 4 and S5†). This was achieved by computing the maximum fingerprint Tanimoto similarity from each molecule containing a given label to each molecule containing any of the 10 most commonly co-occurring labels (with <1000 total abundance). This value was compared to the maximum similarity computed from each molecule containing a given label to a random equal-sized set of molecules to determine significance. This comparison indicated that molecules containing the 10 most commonly co-occurring labels were closer to the given label's molecules in structure space than a random set for 1520 of the 1522 labels (independent *t*-tests per label, false-discovery rate of 5%), meaning that text-based functional relationships align with structural relationships. With the discovery of semantically structured communities, above, this suggests that users can move between labels to identify new compounds and *vice versa* to assess a compound's function.

### 3.5 Functional label-guided drug discovery

**Fig. 5:**
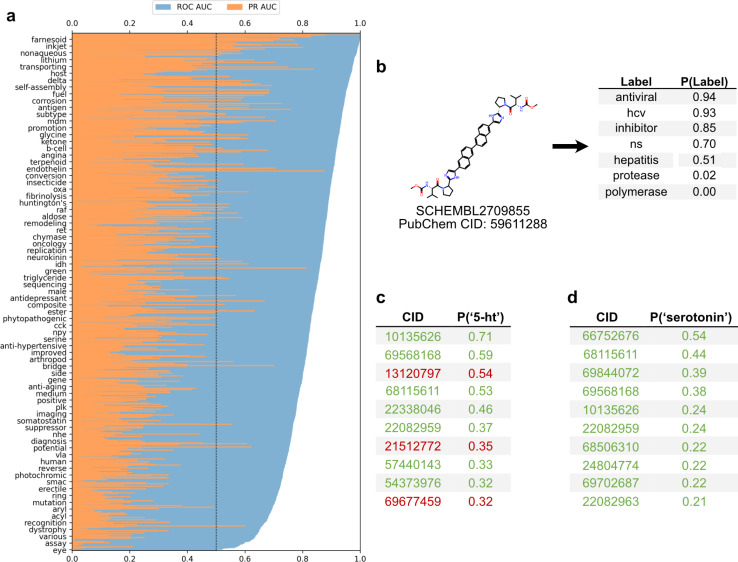
Functional label-guided drug discovery. (a) Test set results from best-performing model that predicts functional labels from molecular fingerprints. Labels sorted by ROC-AUC, showing every 20 labels for clarity. Black line indicates the ROC-AUC random threshold. Average test ROC-AUC and PR-AUC were 0.84 and 0.20, respectively. (b) Model-based comprehensive annotation of chemical function. Shown is a test set molecule patented for hepatitis C antiviral treatment. The highly predicted ‘hcv’, ‘ns’ (nonstructural), and ‘inhibitor’ with the low-predicted ‘protease’ and ‘polymerase’ can be used to infer that the drug acts on NS5A to inhibit HCV replication, revealing a mechanism undisclosed in the patent. (c and d) Functional label-based drug candidate identification, showcasing the top 10 test set molecules for ‘serotonin’ or ‘5-HT’; true positives in green and false positives in red, determined if their associated patents mentioned serotonin or serotonin receptors. The false positives offer potential for drug discovery and repurposing, especially when considering these have patents for related neurological uses (*i.e.*, anti-anxiety and memory dysfunction).

To employ the text-based chemical function landscape for drug discovery, several multi-label classification models were trained on CheF to predict functional labels from daylight molecular fingerprints (Table S7†).^23^ The best performing model was a multi-label logistic regression model on molecular fingerprints that had positive predictive power for 1520/1522 labels and >0.90 ROC-AUC for 433/1522 labels (Fig. 5a). Despite excluding over-patented molecules from the dataset, the CheF-trained model is often able to confidently retrodict their primary functions, giving evidence to our earlier hypothesis (Table S8†).

This model can thus be used to comprehensively annotate chemical function, even when existing annotations are fragmented or incomplete. As an example, for a known hepatitis C antiviral the model strongly predicted ‘antiviral’, ‘hcv’, ‘ns’ (nonstructural) (94%, 93%, 70% respectively) while predicting ‘protease’ and ‘polymerase’ with low confidence (0.02%, 0.00% respectively) (Fig. 5b). The low-confidence ‘protease’ and ‘polymerase’ predictions suggested that the likely target of this drug was the nonstructural NS5A protein, rather than the NS2/3 proteases or NS5B polymerase, a hypothesis that has been validated outside of patents in the scientific literature.^30^

The ability to comprehensively predict functional profiles allows for the discovery of new drugs. For example, the label ‘serotonin’ was used to query the test set predictions, and a ranked list of the 10 molecules most highly predicted for ‘serotonin’ were obtained (Fig. 5c). All ten of these were patented in relation to serotonin: 8 were serotonin receptor ligands (5-HT1, 5-HT2, 5-HT6) and 2 were serotonin reuptake inhibitors. Similarly, the synonymous label ‘5-HT’ was used as the query and the top 10 molecules were again obtained (Fig. 5d). Of these, seven were patented in relation to serotonin (5-HT1, 5-HT2, 5-HT6), four of which were also found in the aforementioned ‘serotonin’ search. The remaining three molecules were patented without reference to the serotonin receptor, but were instead patented for depressant, anti-anxiety, and memory dysfunction relieving effects, all of which have associations with serotonin and its receptor. The identification of known serotonin receptor ligands, together with the overlapping results across synonymous labels, provides an internal validation of the model. Additionally, these search results suggest experiments in which the “mispredicted” molecules may bind to serotonin receptors or otherwise be synergistic with the function of serotonin, thereby demonstrating the practical utility of moving with facility between chemicals and their functions.

To examine the best model's capability in drug repurposing, functional labels were predicted for 3242 stage-4 FDA approved drugs (Fig. S7†).^31^ Of the 16 drugs most highly predicted for ‘hcv’, 15 were approved Hepatitis C Virus (HCV) antivirals. Many of the mispredictions in the top 50 were directly relevant to HCV treatment including 8 antivirals and 8 polymerase inhibitors. The remaining mispredictions included 3 ACE inhibitors and 2 BTK inhibitors, both of which are peripherally associated with HCV through liver fibrosis mitigation and HCV reactivation, respectively.^32,33^ Beyond showing its power, this example suggests that functional label-guided drug discovery may serve as an additional approach for antiviral repurposing which could help contribute to mitigating future pandemics.

## 4. Discussion

While *in silico* drug discovery often proceeds through structural and empirical methods such as protein–ligand docking, receptor binding affinity prediction, and pharmacophore design, we set out to investigate the practicality of methods that leverage the extensive corpus of chemical literature. To do so, we developed an LLM- and embedding-based method to create a Chemical Function (CheF) dataset of 100 K molecules and their 631 K patent-derived functional labels. Over 83% of the functional labels corresponded to distinct clusters in chemical structure space, indicating congruence between chemical structures and individual text-derived functional labels. Moreover, there was a semantically coherent text-based chemical function landscape intrinsic to the dataset that was found to correspond with broad fields of functionality. Finally, it was found that the relationships in the text-based chemical function landscape mapped with high fidelity to chemical structure space (99.9% of labels), indicating approximation to the actual chemical function landscape.

To leverage the chemical function landscape for drug discovery, several models were trained and benchmarked on the CheF dataset to predict functional labels from molecular fingerprints (Table S7†). The top-performing model was utilized for practical applications such as unveiling an undisclosed drug mechanism, identifying novel drug candidates, and mining FDA-approved drugs for repurposing and combination therapy uses. Since the CheF dataset is scalable to the entire 32M + SureChEMBL database, we anticipate that many of these predictions will only get better into the future.

The CheF dataset inherently exhibits a bias toward patented molecules. This implies sparse representation of chemicals with high utility but low patentability and allows for false functional relationships to arise from prophetic claims. Additionally, by restricting the dataset to chemicals with <10 patents, it neglects important well-studied molecules like Penicillin. However, we found the impact of this omission to be negligible (Table S8†). The inclusion of over-patented chemicals, like those in Table S2†, could be accomplished through supplementation from other data sources like PubChem, PubMed, or International Patent Classification categories (Table S1†). These over-patented molecules could also be included through keyword filtering or by only using the most common terms for each molecule. Increasing label quality and ignoring extraneous claims might be achieved through an LLM fine-tuned on high-quality examples or through the organization of functional labels into an ontology. While it is possible that some of the representative terms created with GPT-4 capture hierarchical relationships, it is not guaranteed from the method used herein. Further quality increases may result from integration of well-documented chemical–gene and chemical–disease relationships from PubChem into CheF. As the scope of the manuscript lies with using LLMs to mine functionality from text, we leave dataset merging and supplementation to future work.

The CheF dataset was created from patented molecules. This includes the active molecules responsible for the patent's existence, but also derivatives that may or may not be active. Models trained on the CheF dataset are then learning a coarse-grained map of the chemical function landscape rather than a fine-grained map with activity cliffs. As such, we foresee CheF-trained models being used to annotate broad functionality at a high-level, capturing general chemical trends, rather than providing precise guarantees of activity.

Consideration of ML chemistry dual-use often focuses on the identification of toxic chemicals and drugs of abuse. To test the dual use potential of CheF, functional labels for the chemical weapons VX and mustard gas were predicted from our model and were found to contain no obvious indications of malicious properties. In contrast, drugs of abuse were more easily identifiable, as the development of neurological compounds remains a lucrative objective. 5-MeO-DMT, LSD, fentanyl, and morphine all had functional labels of their primary mechanism predicted with moderate confidence. However, benign molecules also predicted these same labels, indicating that it may be quite challenging to intentionally discover novel drugs of abuse using the methods contained herein.

The analysis herein suggests that a sufficiently large chemical function dataset contains a text-based function landscape that approximates the actual chemical function landscape. Further, we demonstrate one of the first examples of functional label-guided drug discovery, made possible utilizing state-of-the-art advances in machine learning. Models in this paradigm have the potential to automatically annotate chemical function, examine non-obvious features of drugs such as side effects, and down-select candidates for high-throughput screening. Moving between textual and physical spaces represents a promising paradigm for drug discovery in the age of machine learning.

## Data availability

The CheF dataset has been made publicly available under the MIT license at https://doi.org/10.5281/zenodo.8350175. An interactive visualization of the dataset can be found at https://chefdb.app. All code and data used herein may be found at https://github.com/kosonocky/CheF.

## Author contributions

Conceptualization, C. W. K.; methodology, C. W. K.; software, C. W. K. and C. O. W.; validation, C. W. K.; formal analysis, C. W. K.; investigation, C. W. K.; resources, A. D. E., E. M. M., and C. O. W.; data curation, C. W. K.; writing – original draft, C. W. K.; writing – review & editing, C. W. K., A. D. E., E. M. M., and C. O. W.; visualization, C. W. K and C. O. W.; supervision, A. D. E., E. M. M., and C. O. W.; funding acquisition, A. D. E., E. M. M., and C. O. W.

## Conflicts of interest

The authors report no conflict of interest.

## Supplementary Material

DD-003-D4DD00011K-s001

DD-003-D4DD00011K-s002

DD-003-D4DD00011K-s003

DD-003-D4DD00011K-s004

DD-003-D4DD00011K-s005

DD-003-D4DD00011K-s006

DD-003-D4DD00011K-s007

DD-003-D4DD00011K-s008

## References

[cit1] Li Q., Kang C. (2020). Int. J. Mol. Sci..

[cit2] Corso G. , Stärk H., Jing B., Barzilay R. and Jaakkola T., International Conference on Learning Representations, arXiv, 2023, preprint, arXiv:2210.01776v2, 10.48550/ARXIV.2210.01776

[cit3] Trott O., Olson A. J. (2009). J. Comput. Chem..

[cit4] Wu Z., Ramsundar B., Feinberg E. N., Gomes J., Geniesse C., Pappu A. S., Leswing K., Pande V. (2018). Chem. Sci..

[cit5] Yang S.-Y. (2010). Drug Discovery Today.

[cit6] Drachman D. A. (2014). Alzheimer's Dementia.

[cit7] G. O. Consortium (2004). Nucleic Acids Res..

[cit8] Wishart D. S., Girod S., Peters H., Oler E., Jovel J., Budinski Z., Milford R., Lui V. W., Sayeeda Z., Mah R., Wei W., Badran H., Lo E., Yamamoto M., Djoumbou-Feunang Y., Karu N., Gautam V. (2023). Nucleic Acids Res..

[cit9] Degtyarenko K., De Matos P., Ennis M., Hastings J., Zbinden M., McNaught A., Alcantara R., Darsow M., Guedj M., Ashburner M. (2007). Nucleic Acids Res..

[cit10] Edwards C. , Zhai C. and Ji H., in Proceedings of the 2021 Conference on Empirical Methods in Natural Language Processing, 2021, pp. 595–607

[cit11] Li J., Sun Y., Johnson R. J., Sciaky D., Wei C.-H., Leaman R., Davis A. P., Mattingly C. J., Wiegers T. C., Lu Z. (2016). Database.

[cit12] Fu G., Batchelor C., Dumontier M., Hastings J., Willighagen E., Bolton E. (2015). J. Cheminf..

[cit13] Subramanian A., Greenman K. P., Gervaix A., Yang T., Gómez-Bombarelli R. (2023). Digital Discovery.

[cit14] Brown T., Mann B., Ryder N., Subbiah M., Kaplan J. D., Dhariwal P., Neelakantan A., Shyam P., Sastry G., Askell A. (2020). et al.. Adv. Neural Inf. Process. Syst..

[cit15] OpenAI , arXiv, 2023, preprint, arXiv:2303.08774v6, 10.48550/ARXIV.2303.08774

[cit16] Touvron H. , Martin L., Stone K., Albert P., Almahairi A., Babaei Y., Bashlykov N., Batra S., Bhargava P., Bhosale S., et al., arXiv, 2023, preprint, arXiv:2307.09288, 10.48550/arXiv.2307.09288

[cit17] Senger S. (2017). J. Cheminf..

[cit18] Ashenden S. K., Kogej T., Engkvist O., Bender A. (2017). J. Chem. Inf. Model..

[cit19] Kosonocky C. W., Feller A. L., Wilke C. O., Ellington A. D. (2023). Patterns.

[cit20] Martin Y. C., Kofron J. L., Traphagen L. M. (2002). J. Med. Chem..

[cit21] Papadatos G., Davies M., Dedman N., Chambers J., Gaulton A., Siddle J., Koks R., Irvine S. A., Pettersson J., Goncharoff N., Hersey A., Overington J. P. (2016). Nucleic Acids Res..

[cit22] Weininger D. (1988). J. Chem. Inf. Comput. Sci..

[cit23] RDKit: open-source cheminformatics software, RDKit, 2013, https://rdkit.org

[cit24] Kim S., Thiessen P. A., Bolton E. E., Chen J., Fu G., Gindulyte A., Han L., He J., He S., Shoemaker B. A. (2016). et al.. Nucleic Acids Res..

[cit25] Ester M. , Kriegel H.-P., Sander J., Xu X., et al., in Knowledge Discovery and Data Mining, 1996, vol. 96, pp. 226–231

[cit26] Bastian M. , Heymann S. and Jacomy M., in Proceedings of the international AAAI conference on web and social media, 2009, vol. 3, pp. 361–362

[cit27] Maggiora G., Vogt M., Stumpfe D., Bajorath J. (2014). J. Med. Chem..

[cit28] Patterson D. E., Cramer R. D., Ferguson A. M., Clark R. D., Weinberger L. E. (1996). J. Med. Chem..

[cit29] Bajusz D., Rácz A., Héberger K. (2015). J. Cheminf..

[cit30] Ascher D. B., Wielens J., Nero T. L., Doughty L., Morton C. J., Parker M. W. (2014). Sci. Rep..

[cit31] Ochoa D., Hercules A., Carmona M., Suveges D., Gonzalez-Uriarte A., Malangone C., Miranda A., Fumis L., Carvalho-Silva D., Spitzer M. (2021). et al.. Nucleic Acids Res..

[cit32] Corey K. E., Shah N., Misdraji J., Abu Dayyeh B. K., Zheng H., Bhan A. K., Chung R. T. (2009). Liver Int..

[cit33] Mustafayev K., Torres H. (2022). Clin. Microbiol. Infect..

