# Supplementary material for: Mining patents with large language models elucidates the chemical function landscape
Source: Digit Discov. 2024 May 7;3(6):1150–9. doi: 10.1039/d4dd00011k (PMC11167698; doi:10.1039/d4dd00011k)
Supplement: DD-003-D4DD00011K-s001 [file DD-003-D4DD00011K-s001.pdf]

Electronic Supplementary Material (ESI) for Digital Discovery.
This journal is © The Royal Society of Chemistry 2024

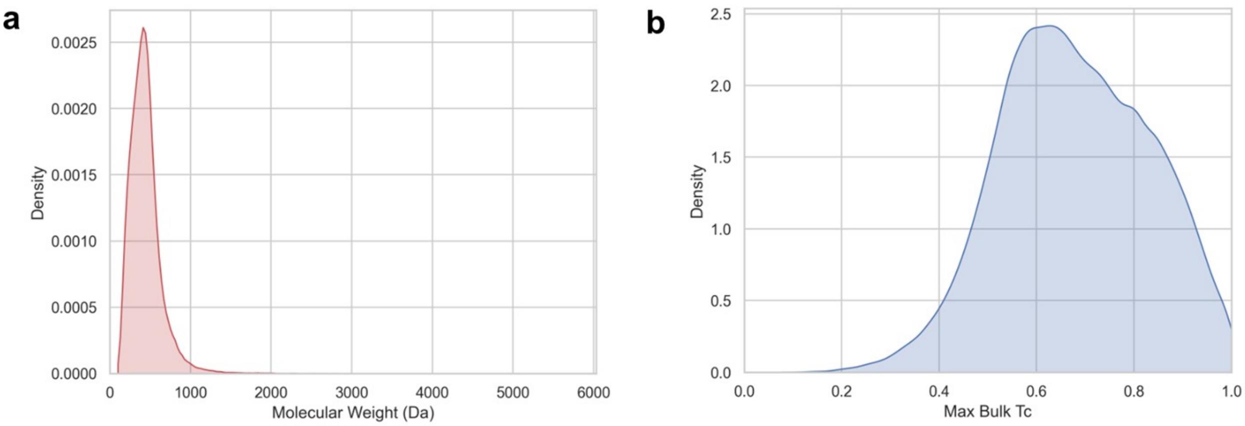

Figure S1. Molecular weight and structural similarity distribution of the CheF dataset. (a) Molecular weight of each molecule in the dataset. Minimum: 100.12 Da; Maximum: 5749.60 Da; Mean: 440.79 Da; Std: 203.96 Da. **(b)** Maximum bulk fingerprint Tanimoto coefficient (Tc) for each molecule in the dataset. Bulk Tc measures how similar a given molecule's structure is to all of the other molecules in the dataset. Max Bulk Tc returns the structural similarity of a molecule to the most structurally similar molecule in the dataset. High Max Bulk Tc indicates redundant structures, mid-low Max Bulk Tc indicates diverse structures. Minimum: 0.076; Maximum: 1.00; Mean: 0.68; Std: 0.15.

Table S1. International Patent Classification (IPC) categories vs ChatGPT-generated functional labels. IPC categories and ChatGPT-generated labels were obtained for SCHEMBL4156630 (PubChem CID: 87628486) from the patent US-2009156572-A1. The IPC categories include structural classifications (e.g., Ortho-condensed systems, Heterocyclic compounds containing) and broad disease classifications (e.g., Centrally acting analgesics, Antidepressants) but miss out on the fine-grained mechanistic terms that the ChatGPT-aided method captures (e.g., Dopamine-Antagonistic, Serotonin Agonist).

**IPC categories**	**ChatGPT-generated functional labels**
C07D471/04 Ortho-condensed systems	Central Nervous System
C07D401/04 Heterocyclic compounds containing two or more hetero rings, having nitrogen atoms as the only ring hetero atoms, at least one ring being a six-membered ring with only one nitrogen atom containing two hetero rings directly linked by a ring-member-to-ring-member bond	Dopamine-Antagonistic
A61P25/00 Drugs for disorders of the nervous system	Dopamine-Agonistic
A61P25/04 Centrally acting analgesics, e.g. opioids	Serotonin Agonist
A61P25/06 Antimigraine agents	Serotonin Antagonist

A61P25/18 Antipsychotics, i.e. neuroleptics; Drugs for mania or schizophrenia	Analgesic
A61P25/20 Hypnotics; Sedatives	
A61P25/24 Antidepressants	
A61P25/26 Psychostimulants, e.g. nicotine, cocaine	
A61P25/28 Drugs for disorders of the nervous system for treating neurodegenerative disorders of the central nervous system, e.g. nootropic agents, cognition enhancers, drugs for treating Alzheimer's disease or other forms of dementia	
A61P25/30 Drugs for disorders of the nervous system for treating abuse or dependence	
A61P43/00 Drugs for specific purposes, not provided for in groups A61P1/00-A61P41/00	
C07D401/14 Heterocyclic compounds containing two or more hetero rings, having nitrogen atoms as the only ring hetero atoms, at least one ring being a six-membered ring with only one nitrogen atom containing three or more hetero rings	

Table S2. Randomly sampling from highly patented molecules would pollute the dataset labels. To determine if random sampling could be an option to include molecules with a high number of patent associations (>10,000 patents per molecule), a random sample of 10 patents from 11 distinct molecules was obtained. These patents were categorized as follows: the patent describes the molecule's function (correct), the patent describes the final product's function of which the linked molecule is an intermediate (intermediate), and the patent is irrelevant to the linked molecule (irrelevant).

Name	PubChem CID	Primary Function	# Patents	Correct	Intermediate	Irrelevant
Carvedilol	152743196	Beta Blocker	25959	3	0	7
Tetrakis(acetyloxy)plumbane	16684437	Catalyst	13113	0	0	10
Inulin	24763	Fiber	69262	2	0	8

3-Hydroxy-2,2-dimethylpropyl methacrylate	83492	Acrylate Monomer	17741	1	1	8
liothyronine	5920	Thyroid Hormone	30503	1	0	9
dioctyltin	27681	Plastic Stabilizer	13255	1	0	9
Amrinone	3698	Cardiac Drug	12935	0	0	10
N-Isopropylmethylamine	78485	Base	13500	0	0	10
NADH	439153	Metabolite	54626	0	0	10
3-propoxypropan-1-ol	61868	Solvent	30689	0	0	10
Trifluorochloromethane	6392	Refrigerant	13712	1	0	9

Table S3. ChatGPT patent summarization method is invariant to patent version. To investigate whether the patent version influences the choice of functional labels extracted by ChatGPT, functional labels from two different versions for five distinct patents were obtained. The generated terms were identical in all five cases, providing evidence that the patent version has minimal, if any, impact on the generated functional labels.

Patent ID & Year	Functional Terms	Patent ID & Year	Functional Terms
US10899724B2 (2016)	fungicides / pesticides / microbiocidal	US11066375B2 (2020)	fungicides / pesticides / microbiocidal
US9067917B2 (2014)	FLAP modulators / Inflammation treatment / Respiratory disorders	US20150246052A1 (2015)	FLAP modulators / Inflammation treatment / Respiratory disorders
US8133870B2 (2005)	anti-viral / anti-cancer	US20120213735A1 (2012)	anti-viral / anti-cancer
US20120225846A1	Inhibition / PASK /	US9675584B2	Inhibition / PASK /

(2012)	Diabetes	(2015)	Diabetes
US7902192B2 (2004)	Inhibitors / P38 / Cytokine	US20110301160A1 (2010)	Inhibitors / P38 / Cytokine

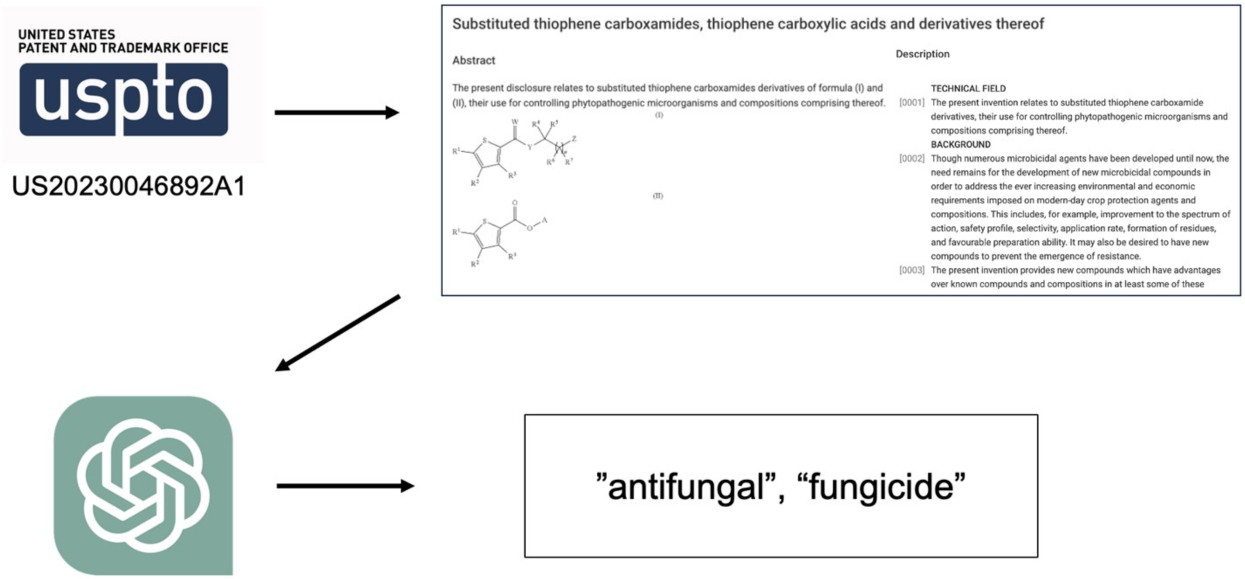

Figure S2: Example of LLM-based chemical function extraction. Patent IDs are used to retrieve the patent title, abstract, and description from Google Patents. ChatGPT is then prompted to extract out the chemical function of the molecule being described by the patent.

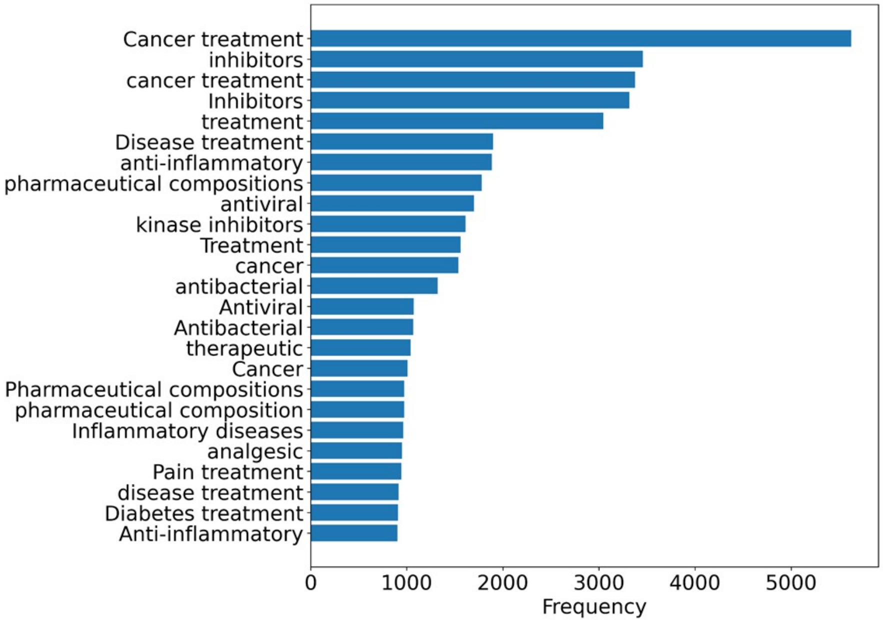

Figure S3: Most frequent patent summarizations. The most frequent patent summarizations do not immediately exhibit any dataset-independent biases. The bias towards broad treatment

terms, such as cancer, antiviral, and analgesic, likely emerged because these are desirable target functions and are thus overrepresented in patents.

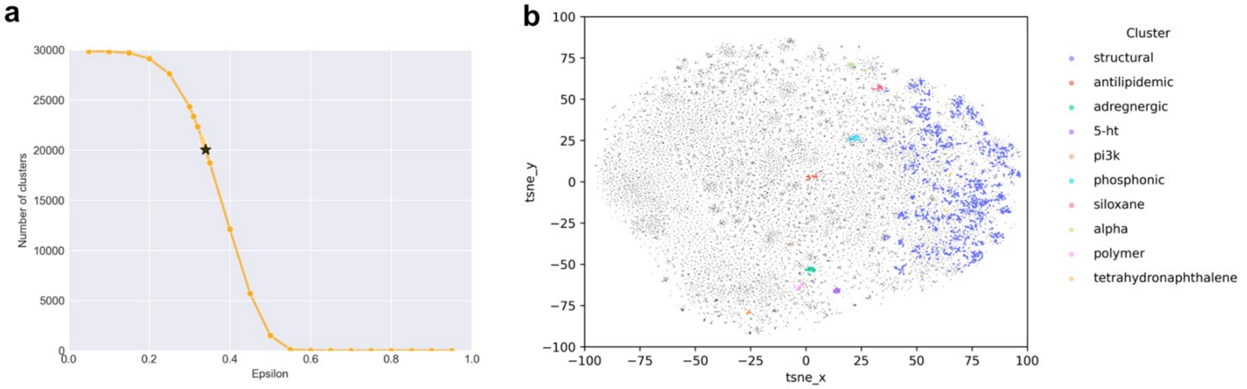

Figure S4: DBSCAN clustering on ada-002 text embeddings reduces the number of labels. **(a)** The optimal DBSCAN epsilon value was defined as the cutoff resulting in the smallest number of clusters without overtly false categories appearing (e.g., merging antiviral, antibacterial, and antifungal). The optimal epsilon was found to be 0.340 for the dataset considered herein (marked by black star), resulting in a consolidation from 29,854 labels to 20,030 clusters. The labels in each cluster were then consolidated with ChatGPT, creating a set of 20,030 labels. **(b)** t-SNE of the ada-002 text embeddings, colored by the top 10 largest clusters. The largest cluster, found to be all IUPAC structural terms, was removed from the dataset to reduce excessive non-generalizable labels.

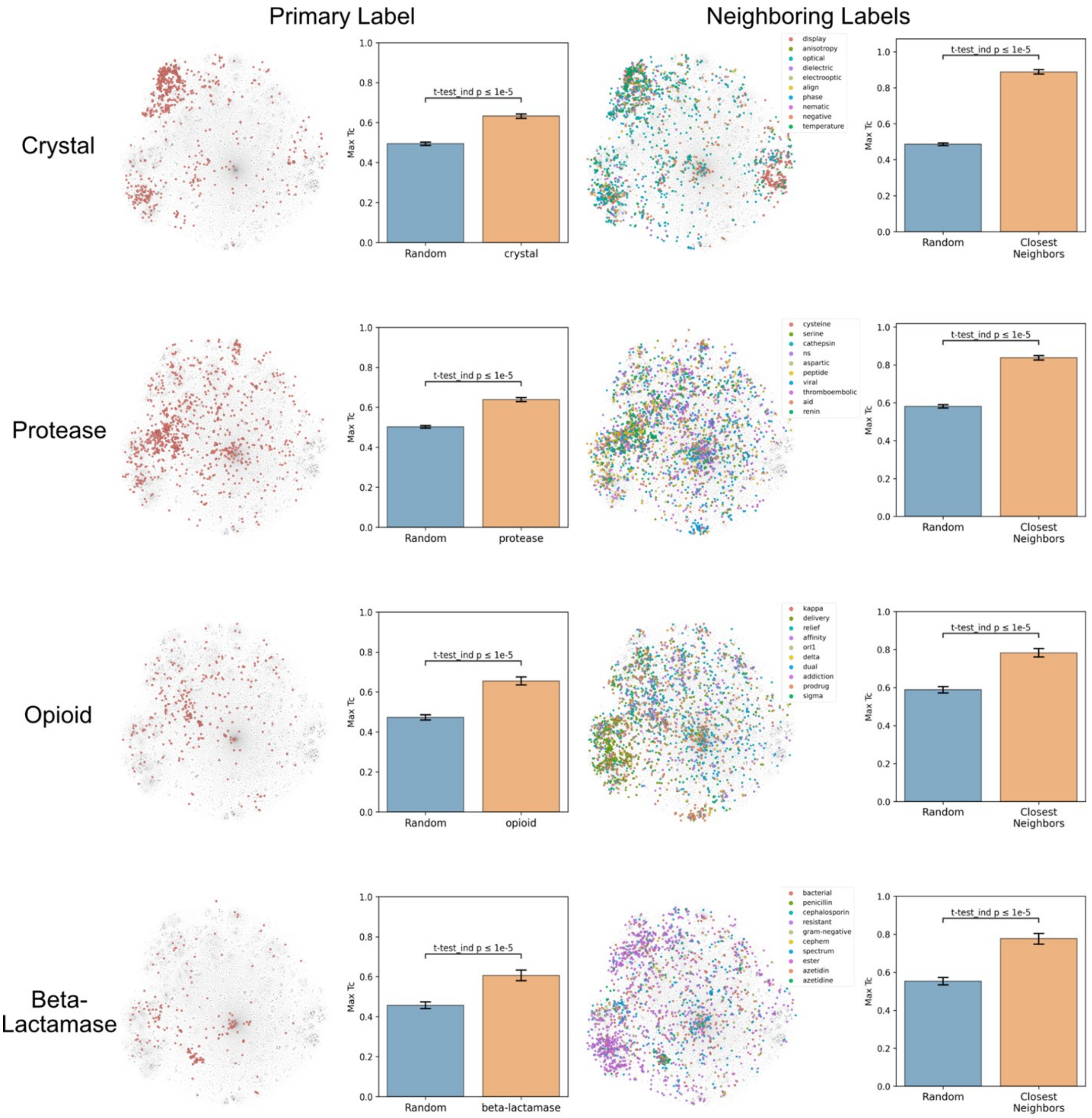

Figure S5: Additional CheF labels and their clusters in structure space. For each of the labels “crystal”, “protease”, “opioid”, and “beta-lactamase”, molecules in the CheF dataset were projected based on molecular fingerprints and colored if the selected label was contained by the molecule’s set of descriptors. To measure degree of clustering for a single label, the max fingerprint Tanimoto similarity from each molecule containing the selected label, to the other molecules containing that label, compared against the max fingerprint Tanimoto similarity for a random subset of molecules of the same size was obtained, whereas to measure the coincidence between the primary and co-occurring labels, the max fingerprint Tanimoto similarity from each molecule containing the primary label to each molecule containing any of the 10 nearest neighbor labels was compared against the max fingerprint Tanimoto similarity to a random subset of molecules of the same size.

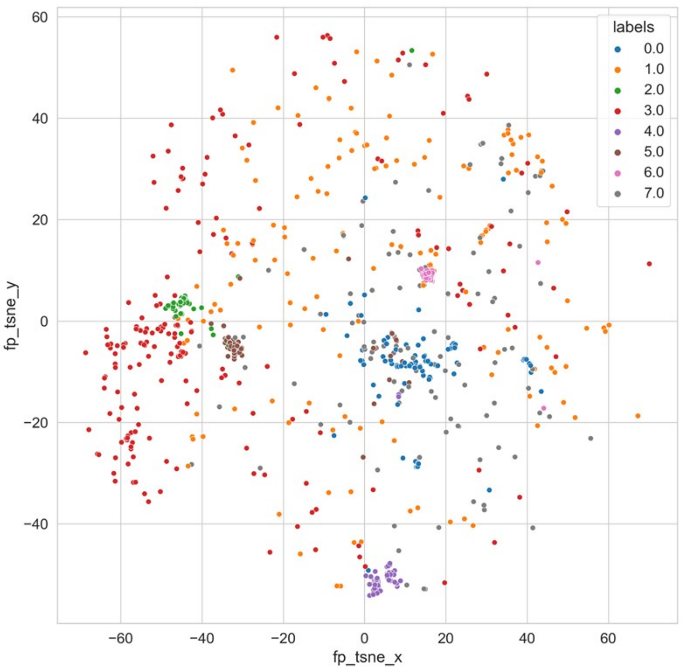

Figure S6: K-means clustering on molecules containing ‘hcv’ elucidates Hepatitis C Virus (HCV) antiviral modalities. The top 20 most frequently occurring labels were obtained for each of 8 clusters to determine their modalities (if applicable). Cluster 4 was the only cluster to contain ‘nucleoside’ (n=65) and ‘nucleotide’ (n=12) in the top 20 labels, indicating this cluster primarily contained HCV antiviral nucleoside derivatives likely inhibiting the NS5B polymerase. Cluster 2 contained ‘protease’ (n=85), ‘peptide’ (n=35), and ‘serine’ (n=15), indicating that this cluster primarily contained peptidomimetic protease inhibitors acting on the NS3 serine protease. Cluster 5 contained ‘protease’ (n=108), ‘macrocyclic’ (n=42), and serine (n=8), indicating that this cluster contained macrocyclic compounds acting likely as NS3 serine protease inhibitors. Cluster 6 contained no specific mechanistic terms, alluding to the possible mechanism of these molecules inhibiting the NS5A protein.

Table S4: GPT-4 graph community summarizations. All labels from the ten most abundant clusters were fed into GPT-4 for categorical summarization. These outputs were verified to be representative of the labels and were further consolidated by the authors into concise categories.

GPT4 Cluster Summary	Label in Graph
Chemical Processes & Reactions, Materials & Substances, Photographic & Printing Processes, Cosmetic & Dermatological Applications, Industrial Manufacturing & Production, Sensory Properties	Material, Industrial, Synthesis, & Dermatology
Antiviral, Cancer, Cellular Processes, Enzymes, Immunology, Oncology, Protein Interactions, Therapy & Drug Development	Antiviral & Cancer
Pain Management, Hormonal Regulation, Gastrointestinal Conditions, Neurological Conditions, Reproductive Health, Obesity Management, Addiction Treatment, Sleep Disorders, Immune Response, Cardiovascular Conditions	Neurological, Hormonal, Gastrointestinal, & Reproductive Health
Chemical Compounds & Materials, Electronic & Optoelectronic Devices, Energy & Efficiency, Light & Emission Properties, Stability & Durability, Quantum & Thermodynamics	Electronic, Photochemical, & Stability
Neurodegenerative Diseases, Inflammatory & Autoimmune Diseases, Respiratory Diseases, Immune Response & Regulation, Enzymes & Mediators, Drug Development & Therapeutics	Neurodegenerative, Autoimmune, Inflammation, & Respiratory
Antibacterial, Antifungal, Antiparasitic, Antimalarial, Antimicrobial, Antiprotozoal, Antitubercular, Insecticide, Herbicide, Fungicide, Pesticide, Acaricide, Nematicidal, Agricultural & Health Protection	Anti-Organism & Agricultural
Drug Development & Delivery, Diagnostic & Monitoring, Gene & Protein Regulation, Epigenetics & Transcription, Immunology & Vaccines	Pharmaceutical Research, Genetic Regulation, Immunology
Neurological & Psychiatric Disorders, Cognitive & Memory Function, Neuropharmacology & Neurotransmission, Mood & Mental Health, Urologic & Sexual Health	Neurological & Urologic

Lipid Metabolism & Cardiovascular Health, Diabetes Management, Organ Health & Protection	Cardiovascular & Lipid Metabolism
Cardiovascular & Renal Disorders, Ion Channels & Transporters, Anesthetics & Muscle Relaxants, Neurological Disorders & Eye Conditions	Cardiovascular, Renal, & Ion Channel

Table S5: Arbitrary 20 CheF labels from each summarized co-occurrence neighborhood. Modularity-based community detection was performed on the CheF co-occurrence graph to obtain 19 distinct communities. The communities appeared to broadly coincide with the semantic meaning of the contained labels, and the largest 10 communities were summarized to a common label. Shown are a random 20 labels from the first five summarized communities.

**Material, Industrial, Synthesis, & Dermatology**	**Antiviral & Cancer**	**Neurological, Hormonal, Gastrointestinal, & Reproductive Health**	**Electrical, Photochemical, & Stability**	**Neurodegenerative, Autoimmune, Inflammation, & Respiratory**
absorb	aid	analgesic	carbazole	activate
acid	antiviral	condition	compound	adhesion
binder	cancer	ligand	expand	alzheimer
care	cell	modulate	life	amyloid
cosmetic	g12	modulator	light	anti-inflammatory
destabilize	hbv	p2x7	material	autoimmune
form	hcv	pain	activated	cox
functional	hepatitis	prophylaxis	amine	disease
ionic	hiv	prostate	anisotropy	elastase
method	inhibit	receptor	aromatic	il-17
modification	inhibition	relief	blue	inflammation
optical	inhibitor	selective	capability	inflammatory
photochromic	integrase	tgr5	characteristic	interferon
plastic	kinase	tract	charge	lung
polymer	kras	treatment	condensed	neurodegenera

				tive
preserve	mapk	various	crystal	neuroinflamma tion
production	nucleoside	5-ht	cyclic	sting
protective	phosphatidyli nositol	addiction	device	airway
sensitivity	phosphorylati on	adrenergic	dielectric	allergic
skin	protein	affinity	diode	allergy

Table S6: Arbitrary 20 CheF labels from each summarized co-occurrence neighborhood. Modularity-based community detection was performed on the CheF co-occurrence graph to obtain 19 distinct communities. The communities appeared to broadly coincide with the semantic meaning of the contained labels, and the largest 10 communities were summarized to a common label. Shown are a random 20 labels from the second five summarized communities.

**Anti-Organism & Agricultural**	**Pharmaceutical Research, Genetic Regulation, Immunology**	**Neurological & Urological**	**Cardiovascular & Lipid Metabolism**	**Cardiovascular, Renal, & Ion Channel**
amide	assay	anticonvulsant	carbonic	cardiovascular
control	bind	cerebral	ischemia	channel
derivative	bromodomain	disorder	level	ion
infection	diagnostic	function	liver	stroke
protection	drug	mitochondrial	prevention	ace
acaricide	potential	neural	reducer	anesthetic
acetic	psma	neuroprotective	reducing	angina
animal	regulator	pde	reduction	angiotensin
anti	sirtuin	schizophrenia	regulate	anti-hypertensive
anti-malarial	targeting	sedative	releasing	blocker
anti-microbial	alter	system	retinoid	calcium

antiparasitic	analog	urologic	vap	cardiac
aryl	atp	anti-psychotic	vascular	cardiotonic
azetidin	atrophy	antidepressant	aldose	circulation
azetidine	bioavailability	antitussive	alleviate	co-transport
bacterial	biological	anxiolytic	antilipidemic	contraction
bactericide	biomarker	brain	blood	diuretic
beta-lactamase	combinatorial	central	cholesterol	failure
bicyclic	cytotoxic	cholinergic	cholesterolemia	heart
bridge	deacetylase	cns	complication	hypertensive

Table S7: Fingerprint models benchmarked on CheF. To assess a baseline benchmark on the CheF dataset of ~100K molecules, several molecular fingerprint-based models were trained on 90% of the training data and evaluated on the 10% test set holdout. Macro average ROC-AUC and PR-AUC was calculated across all 1,522 labels. Logistic regression (LR), random forest classifier (RFC), and a 2-layer multilayer perceptron (MLP) were trained. Parameters for LR and RFC were chosen to be common default values, whereas the MLP layer number and size were chosen through a 5-fold cross validation.

**Model**	**ROC-AUC**	**PR-AUC**
FP + LR	**0.84**	**0.20**
FP + RFC	0.81	0.12
FP + MLP	0.78	0.08

Table S8: Structure-to-function model can retrodict functions of held-out over-patented molecules. Early in the data pipeline, molecules with >10 patents were withheld to avoid data pollution. Shown are the top 10 predicted terms for five arbitrary over-patented molecules withheld from the dataset. Withholding over-patented molecules has apparently minimal detrimental impact on models trained on the CheF dataset as the model appears to infer over-patented function based on its less patented derivatives.

**Penicillin G (antibiotic)**	**Zidovudine (HIV antiviral)**	**Morphine (analgesic)**	**Psilocin (psychedelic)**	**Terephthalate (plastic monomer)**
anti-microbial	nucleoside	opioid	treatment	inhibitor
penicillin	treatment	analgesic	receptor	polymer
derivative	antiviral	receptor	inhibitor	compound
beta-lactamase	acid	treatment	disease	treatment
intermediate	nucleic	pain	5-ht	acid
acid	derivative	antagonist	derivative	derivative
cephalosporin	analog	agonist	disorder	agent
ester	compound	therapeutic	selective	material
agent	dna	derivative	acid	high
inhibitor	rna	inhibitor	agonist	therapeutic

Name	P(hcv)	P(hepatitis)	P(antiviral)	P(ns)	P(protease)	P(polymerase)	P(ace)	P(btk)
DACLATASVIR DIHYDROCHLORIDE	0.95	0.42	0.95	0.84	0.02	0.00	0.00	0.01
DACLATASVIR	0.95	0.42	0.95	0.84	0.02	0.00	0.00	0.01
GRAZOPREVIR	0.94	0.41	0.81	0.59	0.73	0.00	0.00	0.01
BOCEPREVIR	0.91	0.73	0.42	0.03	0.88	0.00	0.00	0.00
PARITAPREVIR	0.83	0.12	0.75	0.67	0.73	0.00	0.00	0.00
SIMEPREVIR	0.83	0.06	0.35	0.09	0.07	0.01	0.00	0.00
SIMEPREVIR SODIUM	0.83	0.06	0.35	0.09	0.07	0.01	0.00	0.00
VOXILAPREVIR	0.75	0.29	0.65	0.24	0.34	0.00	0.00	0.00
SOFOSBUVIR	0.70	0.16	0.86	0.03	0.00	0.23	0.00	0.00
LEDIPASVIR	0.67	0.53	0.86	0.15	0.01	0.00	0.00	0.00
ELBASVIR	0.61	0.58	0.83	0.15	0.01	0.01	0.00	0.00
GLECAPREVIR	0.56	0.15	0.63	0.26	0.23	0.00	0.00	0.01
VELPATASVIR	0.54	0.68	0.81	0.16	0.02	0.00	0.00	0.01
OMBITASVIR	0.41	0.25	0.41	0.02	0.12	0.00	0.00	0.00
NELARABINE	0.33	0.05	0.45	0.00	0.00	0.01	0.00	0.00
PIBRENTASVIR	0.20	0.32	0.35	0.02	0.01	0.00	0.00	0.01
BAZEDOXIFENE	0.10	0.07	0.07	0.01	0.01	0.01	0.00	0.00
BAZEDOXIFENE ACETATE	0.10	0.07	0.07	0.01	0.01	0.01	0.00	0.00
TEGAFUR	0.09	0.02	0.43	0.00	0.01	0.01	0.00	0.00
MICAFUNGIN	0.08	0.04	0.08	0.03	0.19	0.01	0.00	0.00
MICAFUNGIN SODIUM	0.08	0.04	0.08	0.03	0.19	0.01	0.00	0.00
ACYCLOVIR SODIUM	0.08	0.02	0.74	0.00	0.00	0.01	0.00	0.00
RUCAPARIB CAMSYLATE	0.08	0.09	0.10	0.03	0.00	0.01	0.00	0.01
PERINDOPRIL ERBUMINE	0.08	0.08	0.11	0.02	0.06	0.00	0.20	0.00
DARIDOREXANT HYDROCHLORIDE	0.08	0.03	0.07	0.03	0.00	0.00	0.00	0.01
DARIDOREXANT	0.08	0.03	0.07	0.03	0.00	0.00	0.00	0.01
ACALABRUTINIB	0.07	0.06	0.23	0.09	0.03	0.00	0.00	0.78
PERINDOPRIL ARGININE	0.07	0.07	0.11	0.02	0.06	0.00	0.15	0.00
CYTARABINE	0.07	0.01	0.32	0.00	0.00	0.02	0.00	0.00
PERINDOPRIL	0.07	0.07	0.11	0.02	0.07	0.00	0.20	0.00
IDOXURIDINE	0.07	0.01	0.43	0.00	0.01	0.03	0.00	0.00
MARIBAVIR	0.07	0.04	0.20	0.00	0.00	0.01	0.00	0.00
ACALABRUTINIB MALEATE	0.07	0.05	0.24	0.09	0.02	0.00	0.00	0.78
ADENOSINE PHOSPHATE	0.07	0.03	0.17	0.00	0.00	0.02	0.00	0.00
GANCICLOVIR SODIUM	0.07	0.01	0.70	0.00	0.00	0.01	0.00	0.00
REMDESIVIR	0.06	0.07	0.82	0.02	0.01	0.04	0.00	0.00
FLUDARABINE PHOSPHATE	0.06	0.03	0.17	0.00	0.00	0.01	0.00	0.00
GEMCITABINE	0.06	0.02	0.66	0.00	0.00	0.02	0.00	0.00
GEMCITABINE HYDROCHLORIDE	0.06	0.02	0.66	0.00	0.00	0.02	0.00	0.00
URIDINE TRIACETATE	0.06	0.01	0.25	0.00	0.00	0.02	0.00	0.00
RUCAPARIB	0.06	0.10	0.15	0.02	0.00	0.01	0.00	0.01
PENCICLOVIR	0.06	0.03	0.50	0.00	0.00	0.02	0.00	0.00
AMDINOCILLIN PIVOXIL	0.05	0.01	0.04	0.01	0.02	0.01	0.00	0.00
CAPECITABINE	0.05	0.01	0.25	0.00	0.00	0.01	0.00	0.00
FLOXURIDINE	0.05	0.01	0.41	0.00	0.00	0.02	0.00	0.00
BRIVARACETAM	0.05	0.01	0.07	0.01	0.05	0.00	0.00	0.00
DITHIAZANINE	0.05	0.02	0.07	0.00	0.01	0.00	0.00	0.00
BRINZOLAMIDE	0.05	0.08	0.15	0.02	0.02	0.01	0.00	0.00
ENASIDENIB	0.04	0.04	0.05	0.00	0.00	0.00	0.00	0.00
ANIDULAFUNGIN	0.04	0.05	0.12	0.01	0.22	0.00	0.00	0.00

Figure S7: Top 50 FDA-approved drugs predicted to contain the label 'hcv'. The Stage-4 approved drugs list from OpenTargets was passed through the CheF label prediction model. Results were sorted by 'hcv' probability. Relevant and high abundance labels displayed for clarity. Green cells represent approved-use labels from on the OpenTargets page, and red cells represent no approved usage relevant to the given term.
