# Supplementary figures and images for: Mining patents with large language models elucidates the chemical function landscape
Source: Digit Discov. 2024 May 7;3(6):1150–9. doi: 10.1039/d4dd00011k (PMC11167698; doi:10.1039/d4dd00011k)

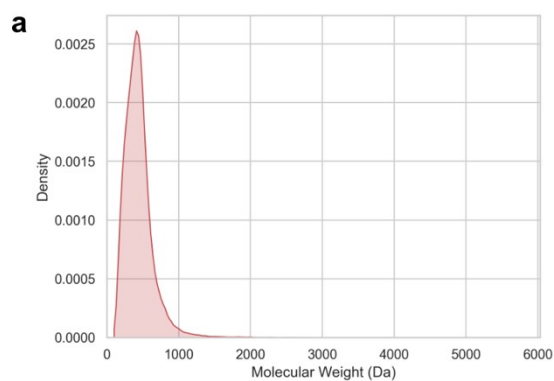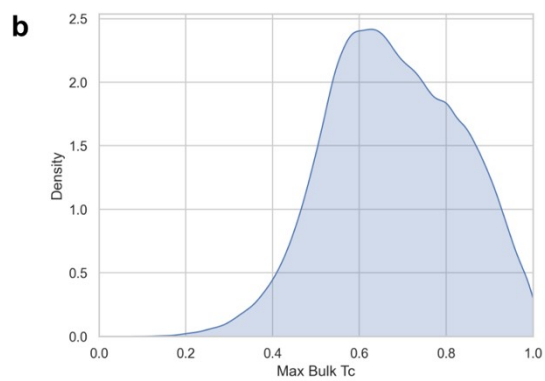

Supplement: DD-003-D4DD00011K-s002 [file DD-003-D4DD00011K-s002.pdf]

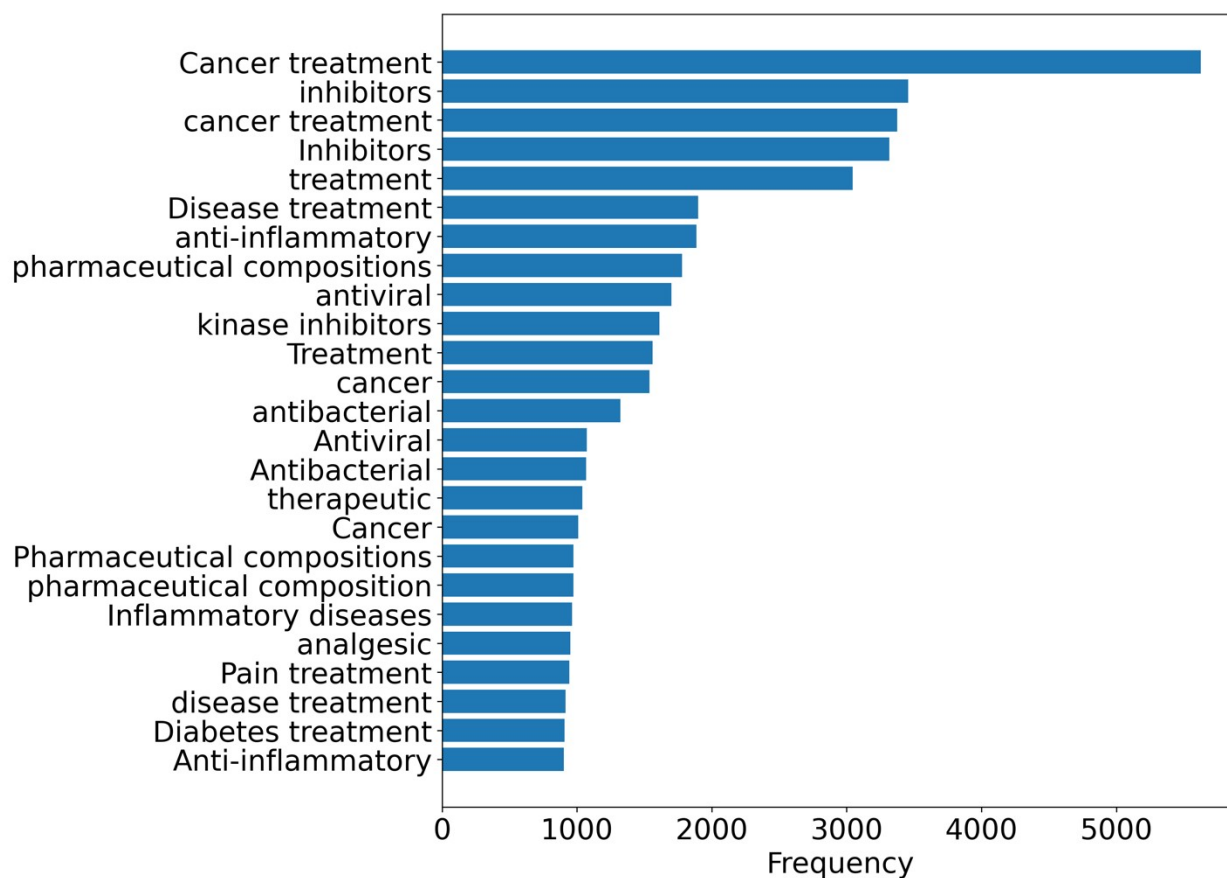

Supplement: DD-003-D4DD00011K-s004 [file DD-003-D4DD00011K-s004.pdf]

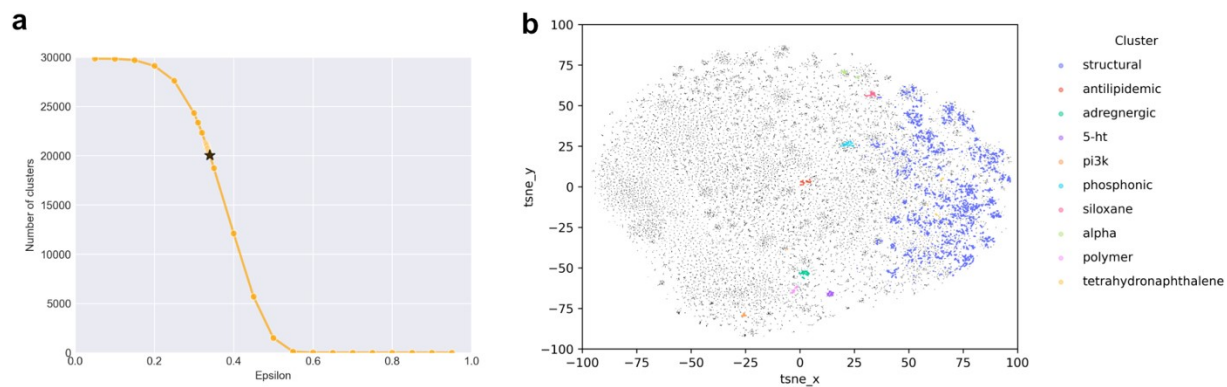

Supplement: DD-003-D4DD00011K-s005 [file DD-003-D4DD00011K-s005.pdf]

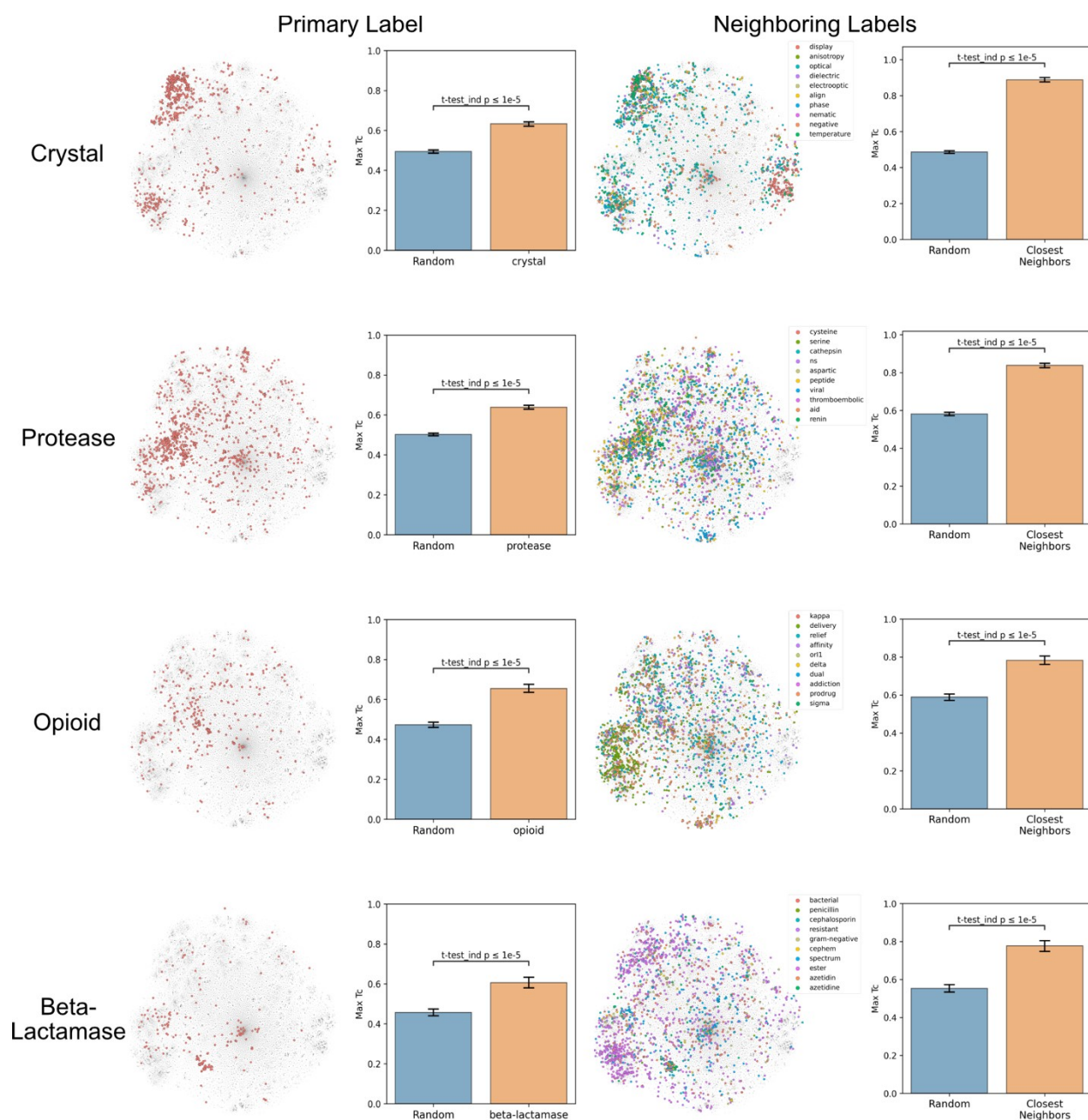

Supplement: DD-003-D4DD00011K-s006 [file DD-003-D4DD00011K-s006.pdf]

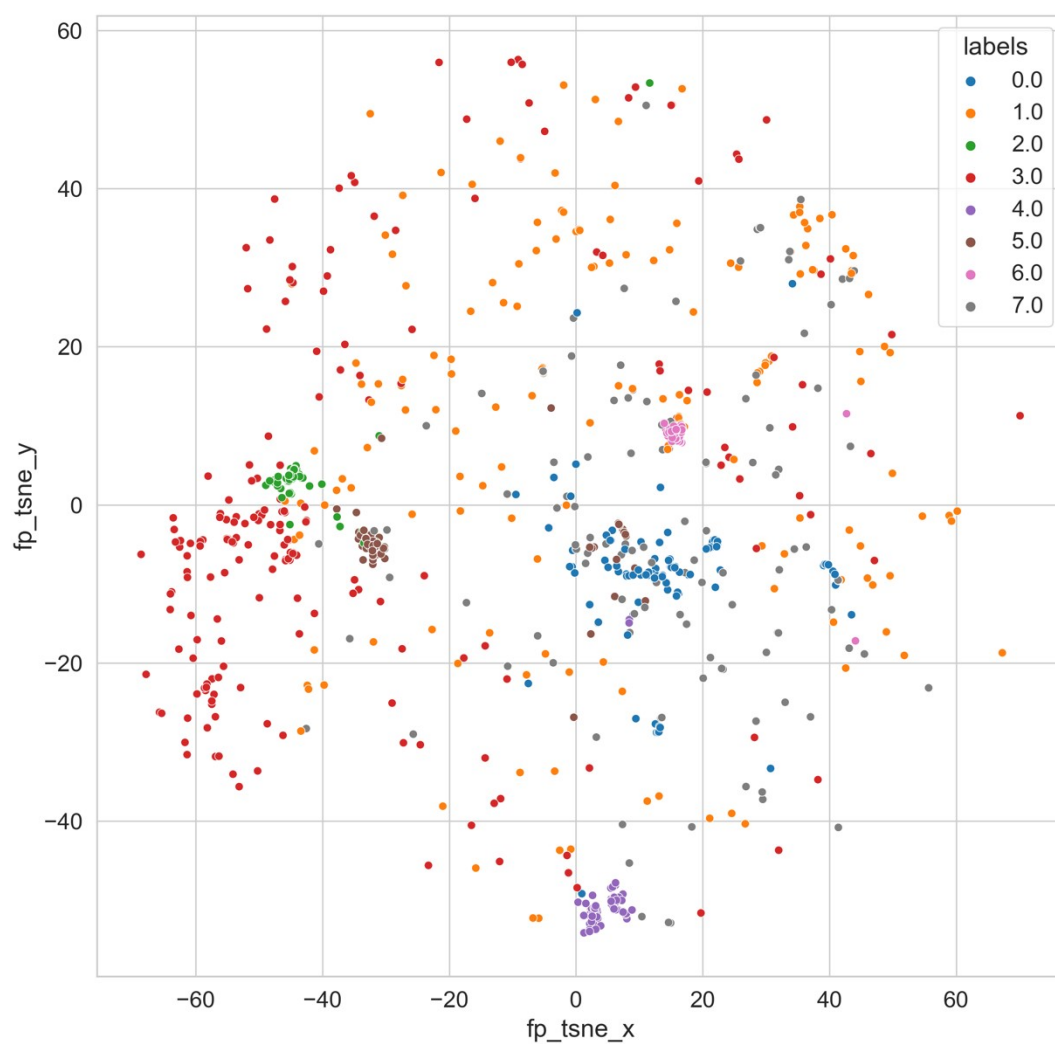

Supplement: DD-003-D4DD00011K-s007 [file DD-003-D4DD00011K-s007.pdf]
