# Supplementary material for: Mining patents with large language models elucidates the chemical function landscape
Source: Digit Discov. 2024 May 7;3(6):1150–9. doi: 10.1039/d4dd00011k (PMC11167698; doi:10.1039/d4dd00011k)

Electronic Supplementary Material (ESI) for Digital Discovery.
This journal is © The Royal Society of Chemistry 2024

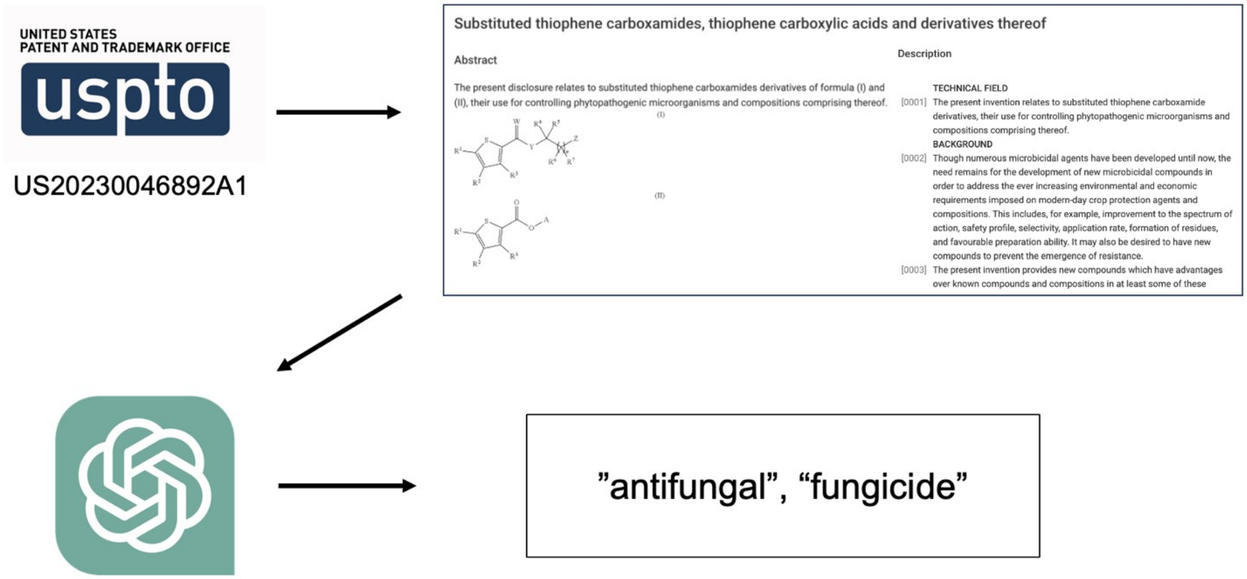

Supplement: DD-003-D4DD00011K-s003 [file DD-003-D4DD00011K-s003.pdf]
