# Supplementary material for: Mining patents with large language models elucidates the chemical function landscape
Source: Digit Discov. 2024 May 7;3(6):1150–9. doi: 10.1039/d4dd00011k (PMC11167698; doi:10.1039/d4dd00011k)
Supplement: DD-003-D4DD00011K-s008 [file DD-003-D4DD00011K-s008.pdf]

Electronic Supplementary Material (ESI) for Digital Discovery.
This journal is © The Royal Society of Chemistry 2024

Name	P(hcv)	P(hepatitis)	P(antiviral)	P(ns)	P(protease)	P(polymerase)	P(hiv)	P(integrase)	P(ace)	P(btk)
DACLATASVIR DIHYDROCHLORIDE	0.96	0.72	0.81	0.95	0.08	0.01	0.07	0.00	0.00	0.01
DACLATASVIR	0.96	0.72	0.81	0.95	0.08	0.01	0.07	0.00	0.00	0.01
GRAZOPREVIR	0.92	0.55	0.56	0.86	0.59	0.00	0.02	0.00	0.00	0.00
BOCEPREVIR	0.87	0.64	0.36	0.15	0.79	0.01	0.02	0.00	0.00	0.00
VELPATASVIR	0.73	0.48	0.50	0.38	0.05	0.01	0.02	0.00	0.00	0.00
PARITAPREVIR	0.70	0.21	0.47	0.62	0.41	0.00	0.02	0.00	0.00	0.00
VOXILAPREVIR	0.69	0.29	0.34	0.61	0.47	0.00	0.02	0.00	0.00	0.00
ELBASVIR	0.66	0.58	0.59	0.45	0.02	0.02	0.03	0.00	0.00	0.00
LEDIPASVIR	0.62	0.39	0.55	0.36	0.03	0.01	0.05	0.00	0.00	0.00
SIMEPREVIR	0.61	0.20	0.26	0.24	0.10	0.01	0.02	0.00	0.00	0.00
SIMEPREVIR SODIUM	0.61	0.20	0.26	0.24	0.10	0.01	0.02	0.00	0.00	0.00
GLECAPREVIR	0.54	0.15	0.28	0.56	0.19	0.00	0.02	0.00	0.00	0.00
SOFOSBUVIR	0.51	0.18	0.72	0.04	0.03	0.06	0.04	0.00	0.00	0.00
OMBITASVIR	0.38	0.17	0.14	0.03	0.06	0.00	0.01	0.00	0.00	0.00
REMDESIVIR	0.23	0.19	0.79	0.02	0.07	0.02	0.20	0.07	0.00	0.00
PIBRENTASVIR	0.22	0.15	0.21	0.13	0.01	0.00	0.04	0.00	0.00	0.00
NELARABINE	0.19	0.08	0.54	0.01	0.00	0.01	0.04	0.00	0.00	0.00
ACALABRUTINIB	0.13	0.09	0.26	0.12	0.01	0.01	0.02	0.00	0.00	0.61
PERINDOPRIL ARGININE	0.13	0.06	0.03	0.06	0.08	0.00	0.00	0.00	0.05	0.00
PERINDOPRIL ERBUMINE	0.12	0.05	0.03	0.08	0.08	0.00	0.00	0.00	0.09	0.00
FOSINOPRIL SODIUM	0.12	0.04	0.15	0.02	0.04	0.01	0.04	0.00	0.03	0.00
FOSINOPRIL	0.12	0.04	0.15	0.02	0.04	0.01	0.04	0.00	0.03	0.00
SPIRAPRIL	0.11	0.05	0.09	0.07	0.05	0.01	0.01	0.00	0.23	0.00
SPIRAPRIL HYDROCHLORIDE	0.11	0.05	0.09	0.07	0.05	0.01	0.01	0.00	0.23	0.00
PERINDOPRIL	0.11	0.04	0.03	0.07	0.08	0.00	0.00	0.00	0.08	0.00
ACALABRUTINIB MALEATE	0.11	0.08	0.24	0.10	0.01	0.01	0.02	0.00	0.00	0.53
ATAZANAVIR	0.11	0.02	0.22	0.02	0.63	0.00	0.15	0.00	0.00	0.00
ATAZANAVIR SULFATE	0.10	0.02	0.21	0.02	0.65	0.00	0.15	0.00	0.00	0.00
BAZEDOXIFENE	0.10	0.06	0.08	0.03	0.01	0.02	0.00	0.00	0.00	0.00
BAZEDOXIFENE ACETATE	0.09	0.06	0.07	0.02	0.01	0.02	0.00	0.00	0.00	0.00
SORIVUDINE	0.09	0.02	0.36	0.01	0.00	0.02	0.03	0.00	0.00	0.00
AZACITIDINE	0.08	0.06	0.17	0.01	0.00	0.01	0.01	0.00	0.00	0.00
MICAFUNGIN	0.08	0.03	0.09	0.06	0.10	0.00	0.01	0.00	0.00	0.00
MICAFUNGIN SODIUM	0.08	0.03	0.09	0.06	0.10	0.00	0.01	0.00	0.00	0.00
RALTEGRAVIR	0.08	0.04	0.48	0.01	0.07	0.00	0.41	0.48	0.00	0.00
RALTEGRAVIR POTASSIUM	0.08	0.04	0.48	0.01	0.07	0.00	0.41	0.48	0.00	0.00
PENTOBARBITAL SODIUM	0.07	0.02	0.04	0.02	0.02	0.01	0.00	0.00	0.00	0.00
METHOHEXITAL SODIUM	0.07	0.02	0.07	0.03	0.03	0.01	0.01	0.00	0.00	0.00
ANIDULAFUNGIN	0.07	0.06	0.09	0.02	0.13	0.00	0.01	0.00	0.00	0.00
TRANDOLAPRIL	0.07	0.03	0.02	0.04	0.16	0.00	0.01	0.00	0.20	0.00
CYTARABINE	0.07	0.02	0.26	0.00	0.00	0.01	0.02	0.00	0.00	0.00
PENCICLOVIR	0.07	0.05	0.40	0.00	0.00	0.01	0.03	0.00	0.00	0.00
GEMCITABINE HYDROCHLORIDE	0.06	0.03	0.39	0.01	0.01	0.01	0.07	0.00	0.00	0.00
GEMCITABINE	0.06	0.03	0.39	0.01	0.01	0.01	0.07	0.00	0.00	0.00
RAMIPRIL	0.06	0.02	0.03	0.03	0.18	0.00	0.01	0.00	0.19	0.00
FLOXURIDINE	0.06	0.02	0.26	0.00	0.01	0.01	0.03	0.00	0.00	0.00
GLASDEGIB	0.05	0.04	0.06	0.04	0.00	0.00	0.02	0.00	0.00	0.00
TENOFOVIR ALAFENAMIDE FUMARATE	0.05	0.03	0.45	0.01	0.00	0.02	0.11	0.00	0.00	0.00
MARIBAVIR	0.05	0.03	0.13	0.01	0.01	0.01	0.01	0.00	0.00	0.00
GLASDEGIB MALEATE	0.05	0.03	0.06	0.03	0.00	0.00	0.02	0.00	0.00	0.00
